# A quantitative method for measuring the relationship between an objective endpoint and patient reported outcome measures

**DOI:** 10.1371/journal.pone.0205845

**Published:** 2018-10-25

**Authors:** Chul Ahn, Xin Fang, Phyllis Silverman, Zhiwei Zhang

**Affiliations:** 1 Center for Devices and Radiological Health, Food and Drug Administration, Silver Spring, Maryland, United States of America; 2 Department of Statistics, University of California, Riverside, CA, United States of America; University of Pisa, ITALY

## Abstract

Patient reported outcome measures (PROMs) become increasingly important for assessing the effectiveness of a drug or medical device. In order for a PROM to be claimed in labeling, the PROM has to be valid, reliable and able to detect a change if the targeted disease status changes. One approach to assess the quality of a patient reported outcome measure (PROM) is to investigate the association between the PROM and an objective clinical endpoint measuring the status of a disease/condition. However, methods assessing the association between continuous and discrete variables are limited, especially for correlated measurements. In this paper, we propose a method to assess such association with any type of samples with or without correlation. The method involves estimating the probability revealing the status of a subject’s disease/condition (called truth thereafter) through the subject’s reported outcomes. The probability is a conditional probability revealing truth given the relative location of the subject’s objective outcome compared to the subject-specific latent threshold in the objective endpoint. A consistent estimator for the probability is derived. The operating characteristics of the consistent estimator are illustrated using simulation. Our method is applied to hypothetical clinical trial data generated for an ophthalmic device as an illustration.

## *1*. Introduction

Patient reported outcome measures (PROMs) have become increasingly important in measuring the effectiveness of a drug or medical device. Between years 1997 and 2002, about 30% of the new drug labels were found to have included patient reported outcomes (PROs) [1]. Later between 2006 and 2010, about 24% of new molecular entities and biologic license applications were granted patient reported outcome (PRO) claims [2]. The authors of this paper also noticed that the PROM claims in approved medical devices had been steadily increasing since 2012. In the meantime, many efforts have been made to advance the use of PROMs in drug or medical device development and regulatory decision making. Recent major challenges were reported from the Food and Drug Administration’s perspective [3]. The National Institutes of Health (NIH) also funded the establishment of a PROM Information System (PROMIS) [4, 5, 6]. Some recent literature focuses on the interpretation of PRO analysis results [7, 8].

In order for a PROM to be claimed in labeling of a drug or medical device, the PROM has to be valid, reliable and able to detect a change if the status of the targeted disease or condition changes [9]. The most frequently and broadly used statistics in a PROM validation such as Pearson and intra-class correlation coefficient (ICC) [10] assess the association among PROM items or between a PROM and other established measurement(s). These correlation coefficients have been used to examine various validities (e.g. construct, convergent/divergent, criterion) of PROMs [10–30]. The correlation coefficients were also used to investigate the PROM’s ability to detect a change [31]. Some authors also used these correlation coefficients to explore the relationship of a PROM with other measurements [32–35].

However, these correlation coefficients (1) may not be appropriate in correlated samples such as repeated measures, (2) may not be reliable for endpoints with different scales (e.g. categorical scale vs. continuous scale), and (3) do not have an intuitive clinical meaning because these coefficients or their changes don’t directly carry a clinical meaning. It is difficult to draw a line for an acceptable association based on these popular coefficients most likely due to the lack of clinical meaning of these correlation coefficients.

The challenge here is to develop a meaningful reliable methodology to measure the relationship between an objective continuous endpoint (*X*) and the dichotomized endpoint (*G*) of an ordinal PROM, and if the association index is strong enough, to use only the PROM to make inference about the effectiveness of the therapy or to use the PROM to support the primary inference in a clinical trial setup. This paper provides such a new meaningful quantitative statistic measuring the conditional association (denoted as *Q* here after) between paired endpoints (*X*, *G*), and a method to translate the ordinary PROM scales to the continuous objective measurement. The use of conditional association is due to the fact that the outcome of *G* is conditional on the outcome of *X*, because the PROM is always administrated after the treatment takes effect. The dichotomized endpoint (*G*) may represent mixed Bernoulli random variables with the same parameter but opposite meaning, which is explained in the method section of this paper.

Section 2 describes the definition of the conditional association parameter *Q*, the data structure used in this paper and how to estimate *Q*. The derivation of the estimator of *Q* is also presented in this section. Section 3 shows simulation results of the estimator ($\hat{Q}$) of *Q* and an application of this new methodology to hypothetical clinical trial data. The discussion and conclusion are presented in Section 4.

## *2*. Methods

This section shows how the parameter *Q* works in assessing the quality of a PROM using repeated measures from a single subject. It starts with minimum notations and theoretical construct of *Q*, followed by the characteristic and estimation procedure of *Q*, and the derivation of the consistent estimator of *Q*. The derivation of the estimator is specifically arranged after introducing the estimation process so that the derivation is more accessible to readers. The section ends with how to obtain the inference for the PROM in multiple subjects.

In general, a single italic lower-case letter represents a nonrandom variable and a single italic upper-case letter represents a random variable unless stated otherwise (such as parameter *Q*). The non-italic PROM_z_ (not a random variable) represents the scale *z* of the unidimensional PROM. *Q*_*iz*_ is the probability of the PROM_z_ revealing the disease status of Subject *i* according to his/her latent minimum objective threshold *a*_*iz*_ given the subject’s objective outcome *x*_*i*_ ≥ *a*_*iz*_ or *x*_*i*_ < *a*_*iz*_. Note: *Q*_*iz*_ is not defined as a random variable and is a parameter to be estimated. The italic *PROM* is the random variable for the subjective PRO measurement, and the italic *PRO* is the realization of the *PROM*. The italic *PRO*_*z*_ represents the patient reported outcome equal to the scale *z* of the PROM. Other notations are defined in Appendix A.

### 2.1 Theoretical construct of parameter *Q*_*iz*_

**Fig 1:**
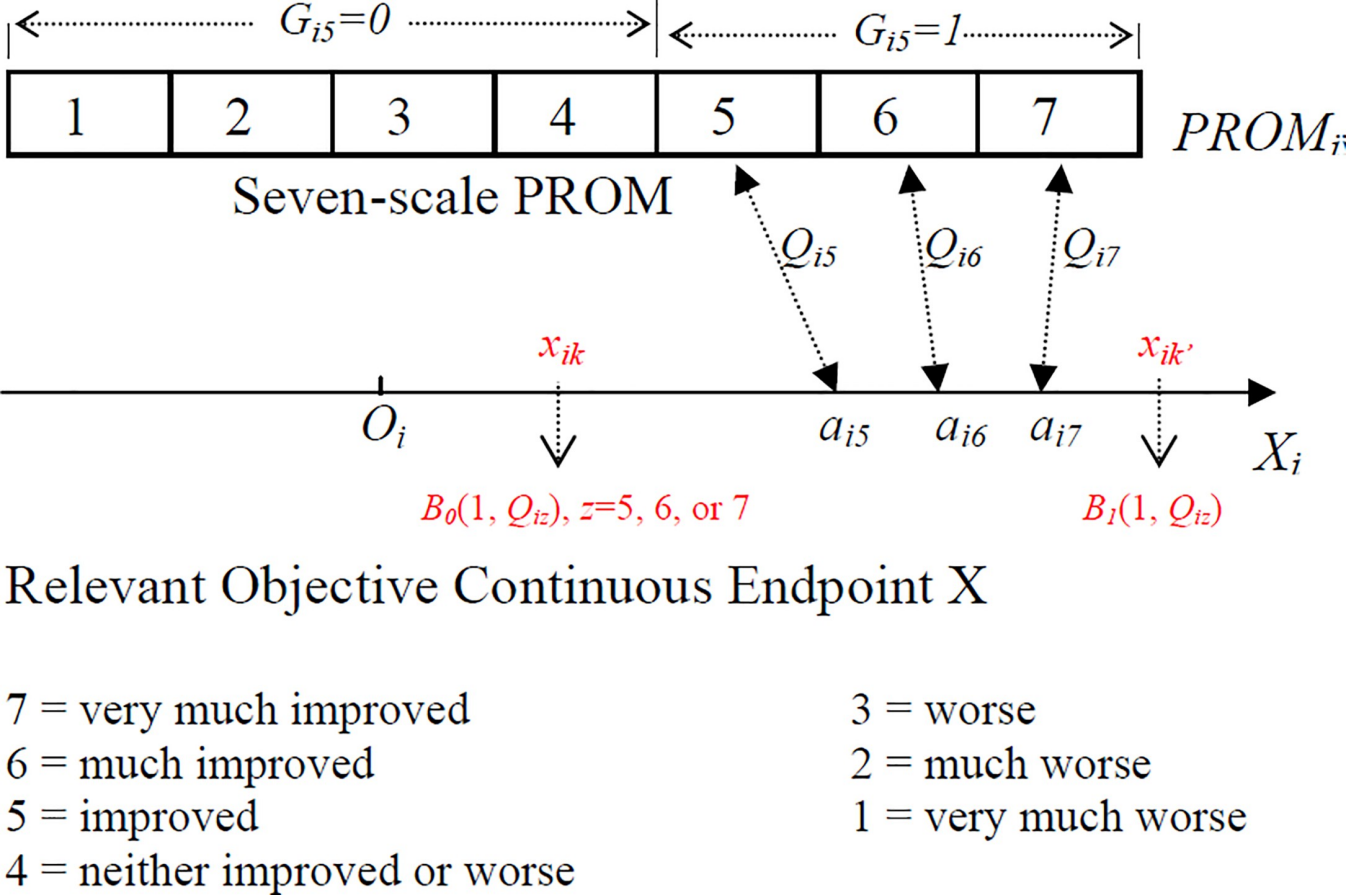
Conditional associations between a *PROM* and a continuous objective efficacy endpoint *X* for subject *i*.

As illustrated in Fig 1 below, the theoretical construct of *Q*_*iz*_ is that there is a latent minimum threshold *a*_*iz*_ of a disease status in terms of the objective disease measurements of Subject *i* which triggers *PRO*_*z*_ (*z* = 1, …, 7 in Fig 1) upon the PROM question according to the association parameter *Q*_*iz*_ given the subject’s objective outcome *x*_*i*_ ≥ *a*_*iz*_. Although the PROM question and scales don’t change with subject, sub-index *i* is used to indicate that the *PROM*_*i*_ is the PROM random variable for Subject *i*, hereafter for clearance and without a loss of generality. Subject *i* will give his/her *PROM*_*i*_ ≥ *z* with probability *Q*_*iz*_ when his/her *x*_*i*_ ≥ *a*_*iz*_, and will give his/her *PROM*_*i*_ < *z* with probability *Q*_*iz*_ when *x*_*i*_ < *a*_*iz*_. Note here, the *PRO*_*i*_ is always dependent on where the *X*_*i*_ is realized relative to the minimum latent threshold *a*_*iz*_.

Fig 1 illustrates the relationship between the continuous objective endpoint *X*_*i*_ (such as increase in hemoglobin count (HC)) and a unidimensional 7-scale *PROM*_*i*_ (such as fatigue improvement). The upper divided rectangular block illustrates a 7-scale unidimensional PROM, and the lower line X illustrates the continuous objective measurement with letter *O* indicating the baseline location of a subject. Each scale of the PROM (such as 5 = improved) for Subject *i* has its own minimum latent objective threshold (such as *a*_*i5*_) pointed by a connecting arrow between the two measurements. The *PROM* will be realized to the *PRO*_*z*_ with probability *Q*_*iz*_ by Subject *i* upon the PROM question if *x*_*i*_ ≥ *a*_*iz*_, which determines the conditional association of the *PROM*_*i*_ with the continuous objective endpoint *X*_*i*_ for Subject *i* at PROM_z_.

Note here, the event of “PROM_z_” revealing the disease status of Subject *i* includes two true events: (1) *PROM*_*i*_ ≥ *z* if *x*_*i*_ ≥ *a*_*iz*_ (as true positive), and (2) *PROM*_*i*_ <*z* if *x*_*i*_ < *a*_*iz*_ (as true negative). We realize that if there is no conditional association between *PROM*_*i*_ and *X*_*i*_ both *Pr*(*PROM*_*i*_ ≥ *z* | *X*_*i*_ ≥ *a*_*iz*_) and *Pr*(*PROM*_*i*_ <*z* | *X*_*i*_ < *a*_*iz*_) are equal to the pure chance rate: 50%. Therefore, we are searching the minimum threshold *a*_*iz*_ in this paper such that Subject *i* will give his/her *PROM*_*i*_ ≥ *z* with probability *Q*_*iz*_ when *x*_*i*_ ≥ *a*_*iz*_; and likewise Subject *i* will give his/her *PROM*_*i*_ < *z* also with probability *Q*_*iz*_ when *x*_*i*_ < *a*_*iz*_. If the probability between the two possible “truths” are not equal, their estimations require many more assumptions (see derivation section for details) and are not considered in this paper. It is also necessary to point out that the two probabilities are not complementary to each other.

### 2.2 Characteristics of parameter *Q*_*iz*_

Parameter *Q*_*iz*_ varies with PROM_z_ and subject based on its definition. Therefore, there is no linear relationship between the *PROM* and the objective endpoint *X* for any subject. For example, *Pr*(*PROM*_*i*_ < 5 | *X*_*i*_ < *a*_*i5*_) may be different from *Pr*(*PROM*_*i*_ < 6 | *X*_*i*_ < *a*_*i6*_); and *Pr*(*PROM*_*i*_ < 5 | *X*_*i*_ < *a*_*i5*_) for Subject *i* may be different from *Pr*(*PROM*_*h*_ < 5 | *X*_*h*_ < *a*_*h5*_) for Subject *h*.

It is obvious that the clinical meaning of *Q*_*iz*_ is inherited from its definition; i.e. **the rate of revealing the truth, conditional on disease status** (the actual disease status of Subject *i* relative to his/her minimum latent objective threshold for PROM_z_). A 50% rate revealing truth is equivalent to the subject flipping a fair coin to determine his/her *PRO*_*z*_ upon the PROM question; thus, this rate of 50% revealing truth indicates that the PROM_z_ is not able to reveal the subject’s disease status. In general, the higher the rate revealing truth is, the better the quality of the PROM_z_ is. This is because the higher rate indicates a higher probability of the PROM_z_ to reveal a subject’s disease status upon the PROM question.

The use of *Q*_*iz*_ to reveal the actual status of a subject’s disease has not been discussed in literature. Rasch promoted a probability model for a true positive response [36]. However, because a negative agreement was not considered, the Rasch positive probability did not measure the probability of revealing truth from a PROM. Our approach is related to latent variable models for similar problems [37, 38] in the sense that *a*_*iz*_ can be regarded as a latent variable. On the other hand, we do not assume a particular distribution for *a*_*iz*_, which makes our approach different from most latent variable models. It is also noteworthy to know that *Q*_*iz*_ is also measuring an indirect agreement between a continuous endpoint and a dichotomized version of an ordinal endpoint. Most traditional methodologies for measuring agreement as described in [39] are developed for two measures of the same type: both categorical or both continuous endpoints. In the case of different types of endpoints, ranks within each endpoint will replace the original values to make the two endpoints the same type (such as Spearman CC). In addition, the estimation of *Q*_*iz*_ (1) can be applied to correlated data, (2) takes into consideration the uncertainty of the “gold standard” and involves a series of 2-by-2 tables in order to select one for the estimate (see the toy example below). Therefore, *Q*_*iz*_ can be also viewed as a new agreement statistic between a continuous endpoint and a binary endpoint with or without correlation among samples.

### 2.3 Data and corresponding random variables

The data considered in this paper consist of pairs of observations (*x*_*ik*_, *g*_*ik*_) for Subject *i* at clinical visit *k*, where *k =* 1, *…*, *t*. This *x*_*ik*_ is a continuous outcome representing disease status and could be the value at visit *k* or the change from baseline to visit *k*, such as the change in hemoglobin count from baseline. The outcome *g*_*ik*_ is the dichotomized version of the collected *PRO*s at visit *k*, such as *g*_*ik*_ = 1 if the *PROM*_*i*_ ≥ 5 and *g*_*ik*_ = 0 otherwise. The change from baseline in the *PROM*_*i*_ is not considered here, because (1) each latent threshold of a PROM_z_ is corresponding to the PROM_z_ itself instead of its change, and (2) a change in *PRO*s from baseline does not carry the same clinical meaning, which depends on the baseline *PRO*s. For example, in a 7-point scale *PROM*_*i*_ shown in Fig 1, a change in one PROM unit from “much worse” to “worse” may not be meaningful to a subject, while a change in one PROM unit from “neither” to “improved” carries clinical meaning to the subject.

**Fig 2:**
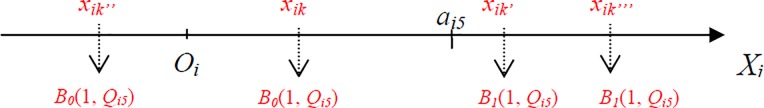
The *g*_*ik*_ is from two Bernoulli random variables with same parameter but opposite meaning depending where the *X*_*ik*_ is realized: *x*_*ik*_ < *a*_*iz*_ or ≥ *a*_*iz*_.

The corresponding random variables are denoted as (*X*_*ik*_, $G_{ik}^{1}$ or $G_{ik}^{0}$). The $G_{ik}^{1}$ is the Bernoulli random variable (*B*_*1*_(1, *Q*_*iz*_)) with probability *Q*_*iz*_ to be 1 when *x*_*ik*_ ≥ *a*_*iz*_, and $G_{ik}^{0}$ is the Bernoulli random variable (*B*_*0*_(1, *Q*_*iz*_)) with parameter *Q*_*iz*_ to be 0 when *x*_*ik*_ < *a*_*iz*_. In other words, upon the PROM question, Subject *i* will give his/her *g*_*ik*_ = 1 (positive) with probability *Q*_*iz*_ when his/her *x*_*ik*_ ≥ *a*_*iz*_, and will give his/her *g*_*ik*_ = 0 (negative) with probability *Q*_*iz*_ when his/her *x*_*ik*_ < *a*_*iz*_ as illustrated in Fig 2 below.

### 2.4 Estimation of *Q*_*iz*_

This subsection shows how to estimate *Q*_*iz*_ using a toy example. The derivation of the estimator of *Q*_*iz*_ can be found in next subsection. In order to estimate *Q*_*iz*_, it is necessary to first search *a*_*iz*_. Because the *a*_*iz*_ is the minimum latent threshold in the objective measurement for the PROM_z_, the search for *a*_*iz*_ can be done using a pre-selected set of values {*a*_*j*,_ *j =* 1, …, *m*} between the possible minimum objective measurement and the maximum objective measurement based on the current medical knowledge for the entire target population (such as normal range of human hemoglobin count). The pre-selected value *a*_*j*_ is not meant to be random, but rather fixed and ideally pre-determined before the realization of *X*_*ik*_. For example, the normal range of human blood hemoglobin concentration can be determined from 5g/dL to 20g/dL so that *a*_*iz*_ is believed to be included in the range for any subject; if the increasing step is 1g/dL between *a*_*j*_ and *a*_*j+1*_, then number of searching points, *m*, is equal to 16 in this case. The magnitude of the increasing step is determined by how precise the *a*_*iz*_ is expected to be. Again, this searching set is not considered random because it doesn’t change with study or subject and may not be changed for decades, such as the normal range of human blood pressures.

**Table 1** shows a toy example of how to estimate *Q*_*iz*_. Note here, the number of searching points *m* need not necessarily be equal to the number (*t*) of clinical visits although we do so for illustration purpose. At each *a*_*j*_, the outcome *x*_*ik*_ (*k* = 1, …, *t*) is compared to *a*_*j*_ one at a time. Then the number of potential true positive (*TP*) and the number of potential true negative (*TN*) responses can be summarized per **Table 2**. For example, in the 1^st^ data row of **Table 1** there are 9 *x*_*i*_ ≥ 5.0 (positive) and only 6 *g*_*i*_ equal to one (PRO positive), therefore the *TP* is equal to 6 (see next paragraph for more details). The total number of such 2-by-2 tables is equal to *m*, as the total number of distinct *a*_*j*_ is *m*. The derivation in next subsection shows that the maximum of *R*_*ij*_ *= (TP+TN)*_*ij*_*/t* is a consistent estimator of *Q*_*iz*_.

**Table 1:** Estimate of *Q*_*iz*_ based on 9 pairs of repeated outcomes (*x*_*ij*_, *g*_*ij*_) from subject *i*.

Samples	*a*_*j*_	TP	TN	FP	FN	*TP+TN*	*R*_*ij*_
(5, 0), (**7, 0**), (9, 0), (11, 1), (12, 1), (13, 1), (14, 1), (15, 1), (16, 1)	5.0	6	0	3	0	6	0.67
	6.0	6	1	2	0	7	0.78
	8.0	6	2	1	0	8	0.89
	**10.0**	**6**	**3**	**0**	**0**	**9**	**1.00**
	**11.0**	**6**	**3**	**0**	**0**	**9**	**1.00**
	12.0	5	3	0	1	8	0.89
	13.5	3	3	0	3	6	0.67
	15.0	2	3	0	4	5	0.56
	16.0	1	3	0	5	4	0.44
(5, 0), (**7, 1**), (9, 0), (11, 1), (12, 1), (13, 1), (14, 1), (15, 1), (16, 1)	5.0	7	0	2	0	7	0.78
	**6.0**	**7**	**1**	**1**	**0**	**8**	**0.89**
	8.0	6	1	1	1	7	0.78
	**10.0**	**6**	**2**	**0**	**1**	**8**	**0.89**
	**11.0**	**6**	**2**	**0**	**1**	**8**	**0.89**
	12.0	5	2	0	2	7	0.78
	13.5	3	2	0	4	5	0.56
	15.0	2	2	0	5	4	0.44
	16.0	1	2	0	6	3	0.33

Note: First sample shows: ${\hat{Q}}_{iz}=1.00$, and the corresponding estimate of *a*_*iz*_ = 10.5

Second sample shows: ${\hat{Q}}_{iz}=0.89$, and the corresponding estimate of *a*_*iz*_ = 10

**Table 2:** Number of cell count at *a*_*j*_ (*j =* 1, …, *m*) for subject *i* and PROM_z_.

Objective efficacy outcome	Dichotomized PRO at PROM_z_	
	PRO Positive	PRO Negative
≥ *a*_*j*_	# of Potential Ture Positive (TP)	# of Potential False Positive (FP)
< *a*_*j*_	# of Potential False Negative (FN)	# of Potential Ture Negative (TN)

Table 1 shows how to use the pre-determined set of *a*_*j*_ (*j =* 1, …, *m*) to calculate *R*_*ij*_ at each *a*_*j*_ based on two sets of 9 pairs of observations (*x*_*i1*_, *g*_*i1*_) … (*x*_*i9*_, *g*_*i9*_) from Subject *i*. The only difference between the two sets of samples is the different values in the 2^nd^ binary outcome *g*_*i2*_ (0 vs. 1). If the *PRO*_*i*_ is positive, *g*_*ik*_ = 1; otherwise *g*_*ik*_ = 0. The pre-determined set of *a*_*j*_ (*j =* 1, …, *9*) is listed in the 2^nd^ column of Table 1. At each *a*_*j*_, one can compare the 9 objective outcomes (*x*_*i1*_, …, *x*_*i9*_) to *a*_*j*_ one at a time, and obtain the numbers of potential TP, FP, TN, FN per **Table 2** above. Thus, each data row of Table 1 displays the four statistics TP, FN, FP, and TN, corresponding to *a*_*j*_. The estimate of *Q*_*iz*_ for Subject *i* at the PROM_z_ is the maximum of *R*_*ij*_. In this paper, if there are multiple tied maximums of *R*_*ij*_ the median of the corresponding *a*_*j*_ is used as an estimate of *a*_*iz*_. This is because at each maximum of *R*_*ij*_, the corresponding *a*_*j*_ could be an estimate of *a*_*iz*_.

### 2.5 Derivation of the estimator of *Q*_*iz*_

As illustrated in Fig 1 above, the *Q*_*iz*_ doesn’t change its magnitude as long as *x*_*i*_ ≥ *a*_*iz*_ or *x*_*i*_ < *a*_*iz*_ although *Q*_*iz*_ changes its meaning from conditional true positive rate (when *x*_*i*_ ≥ *a*_*iz*_) to conditional true negative rate (when *x*_*i*_ < *a*_*iz*_). This is a reasonable setup because the event of *PROM*_*i*_ ≥ *z* is a composite event including *PRO*_*iz*_, *PRO*_*iz+1*_, etc. For example, the event *PROM*_*i*_ ≥ 5 includes *PRO*_*i*_ = 5, 6, or 7. When *x*_*i*_ is far above *a*_*iz*_, Subject *i* may just give a higher *PRO*_*i*_ (say 7) and this event counts as one event of *PROM*_*i*_ ≥ 5. This illustrates the fact that *Q*_*iz*_ can be independent of the distance between *x*_*i*_ and *a*_*iz*_. Because we search *a*_*iz*_ such that *Pr*(*PROM*_*i*_ ≥ *z* | *X*_*i*_ ≥ *a*_*iz*_) = *Pr*(*PROM*_*i*_ <*z* | *X*_*i*_ < *a*_*iz*_) and *Q*_*iz*_ doesn’t change its magnitude as long as *x*_*i*_ ≥ *a*_*iz*_ or *x*_*i*_ < *a*_*iz*_., **we define *Q***_***iz***_ ***= Pr*(*PROM***_***i***_ **≥ *z* | *X***_***i***_ **≥ *a*, ∀ *a*≥ *a***_***iz***_**) = *Pr*(*PROM***_***i***_ **<*z* | *X***_***i***_ **< *b*,** ∀***b < a***_***iz***_**) (**see Fig 2 for the illustration), **where *a* and *b* are two arbitrary values in the objective measurement. Note here, although the clinical meaning of *Q***_***iz***_ **changes from conditional positive rate to conditional negative rate according to *x***_***i***_ ***≥ a***_***iz***_ ***or x***_***i***_ ***< a***_***iz***_**, the magnitude of *Q***_***iz***_ **doesn’t change. This implies that the magnitude of *Q***_***iz***_ **doesn’t change with any subset of *X***_***i***_ ***≥ a***_***iz***_ ***or X***_***i***_ ***< a***_***iz***_**. In order to reflect the setup and the meaning of *Q***_***iz***_**, we use *a* and *b* here to indicate that *Q***_***iz***_ **does not change its magnitude with any subset in *X***_***i***_ ***≥ a***_***iz***_ ***or X***_***i***_ ***< a***_***iz***_.

Also, the derivation of the *Q*_*iz*_ estimator doesn’t assume independence among *X*_*i1*_, …, *X*_*it*_. The cumulative distribution function of *X*_*i1*_ is denoted as *F*_*i1*_. Because the *x*_*i1*_ is obtained in the 1^st^ clinical visit before the realization of *X*_*i2*_,…, *X*_*it*_, the cumulative distribution function of *X*_*ik*_ (denoted as *F*_*ik*_, *k*>1) is the marginal cumulative distribution function, which can be obtained by integrating out *X*_*i1*_, …, *X*_*ik-1*_ from the joint distribution *F*_*Xi1*, …, *Xik*_ for Subject *i*. The use of general form of *F*_*ik*_ in the derivation takes into consideration the correlated samples. The joint distribution *F*_*Xi1*, …, *Xik*_ applies to random variables with or without correlation. Therefore, the *X*_*ik*_ (*k* = 1, …, *t*) are not assumed independent to each other and each *X*_*ik*_ has a different marginal distribution.

The derivation of the estimator of *Q*_*iz*_ starts with the probability of getting *TN* and *TP* at Visit *k*, which are presented in Expressions (1)–(4) below:

- When *a*_*j*_ *< a*_*iz*_:

$$\begin{array}{l} {Pr}_{ijk}\left(TN\right)=Pr\left(X_{ik}<a_{j}\;\mathrm{and}\;G_{ik}=0\right)\\ =Pr\left(X_{ik}<a_{j}\right)Pr\left(G_{ik}=0|X_{ik}<a_{j}\right)\\ =F_{ik}\left(a_{j}\right)Pr\left(G_{ik}=0|X_{ik}<a_{j}\right)\\ =F_{ik}\left(a_{j}\right)Pr\left(G_{ik}=0|X_{ik}<a_{j}<a_{iz}\right)\\ =F_{ik}\left(a_{j}\right)Q_{iz} \end{array}$$ (1)

With same argument, one can have the following:

$$\begin{array}{c} Pr_{ijk}\left(TP\right)=Pr\left(a_{j}<X_{ik}<a_{iz}\;\mathrm{and}\;G_{ik}=1\right)+Pr(a_{iz}<X_{ik}\;\mathrm{and}\;G_{ik}=1)\\ =\left[F_{ik}\left(a_{iz}\right)-F_{ik}\left(a_{j}\right)\right]{\bar{Q}}_{iz}+{\bar{F}}_{ik}\left(a_{iz}\right)Q_{iz} \end{array}$$ (2)

- When *a*_*j*_ ≥ *a*_*iz*_:

$$\begin{array}{c} Pr_{ijk}\left(TN\right)=Pr\left(X_{ik}<a_{iz}\;\mathrm{and}\;G_{ik}=0\right)+Pr(a_{iz}<X_{ik}<a_{j}\;\mathrm{and}\;G_{ik}=0)\\ =F_{ik}\left(a_{iz}\right)Q_{iz}+\left[F_{ik}\left(a_{j}\right)-F_{ik}\left(a_{iz}\right)\right]{\bar{Q}}_{iz} \end{array}$$ (3)

$${Pr}_{\mathrm{ijk}}\left(TP\right)=Pr\left(a_{j}<X_{ik}\;\mathrm{and}\;G_{ik}=1\right)={\bar{F}}_{ik}(a_{j})Q_{iz}$$ (4),

where ${\bar{Q}}_{iz}=1-Q_{iz},\;{\bar{F}}_{ik}=1-F_{ik}$.

Consequently, the expectation of *TP*+T*N* can be shown in Expressions (5) and (6), where *E* is the expectation operator.

- When *a*_*j*_ *≤ a*_*iz*_:

$$E_{ij}(TP+TN)=\sum_{k=1}^{t}\left\{[F_{ik}(a_{j})+{\bar{F}}_{ik}(a_{iz})]Q_{iz}+[F_{ik}(a_{iz})-F_{ik}(a_{j})]{\bar{Q}}_{iz}\right\}$$ (5)

- When *a*_*j*_ *> a*_*iz*_:

$$E_{ij}(TP+TN)=\sum_{k=1}^{t}\left\{[{\bar{F}}_{ik}(a_{j})+F_{ik}(a_{iz})]Q_{iz}+[F_{ik}(a_{j})-F_{ik}(a_{iz})]{\bar{Q}}_{iz}\right\}$$ (6)

If *a*_*j*_ is equal to *a*_*iz*_, both expressions (5) and (6) are reduced to *tQ*_*iz*_. Therefore, *R*_*ij*_ *= (TP+TN)/t* is an unbiased estimator of *Q*_*iz*_ **only if** *a*_*j*_ = *a*_*iz*_, and *TP+TN* follows the binomial distribution when *a*_*j*_ = *a*_*iz*_ because its expectation follows the expectation of the binomial random variable (*tQ*_*iz*_). We further notice that *E*_*ij*_ *(TP+TN)* obtains its maximum at *a*_*iz*_ when *Q*_*iz*_ > 0.5 (i.e. $Q_{iz}-{\bar{Q}}_{iz}>0$) based on the sign of the derivative of *E*_*ij*_ *(TP+TN)* with respect to *a*_*j*_. When *Q*_*iz*_ > 0.5, the derivative of *E*_*ij*_ *(TP+TN)* is positive at the left of *a*_*iz*_ (see Expression 5), and becomes negative at the right of *a*_*iz*_ (see Expression 6). Therefore, *E*_*ij*_ *(TP+TN)* not only reaches its maximum at *a*_*iz*_, but also becomes *tQ*_*iz*_. This is why the unbiased estimate of *Q*_*iz*_ is chosen as the maximum of *R*_*ij*_. Similarly, *E*_*ij*_ *(TP+TN)* obtains its minimum at *a*_*iz*_ when *Q*_*iz*_ < 0.5.

In practice, it is reasonable to assume that a PROM has a non-negative association with the objective endpoint because it is obvious to see a potential direction of the PROM. If a negative association is expected, one can transform the objective outcome in order to have a non-negative association. For the example of a negative associate, if the PROM is the price satisfaction survey and the continuous objective endpoint is the cost of medical expense; then one can transform the cost by multiplying “-1” so that the higher negative cost (smaller cost) is in positive direction. Therefore, *Q*_*iz*_ can be assumed to be ≥ 0.5. If *Q*_*iz*_ = 0.5 (pure chance), this indicates that the PROM_z_ may not be able to reveal the truth; consequently, there is no conditional association between the *PROM* and the objective measurement *X* at PROM_z_. This is because *Q*_*iz*_ is defined as the probability revealing truth at PROM_z_; *Q*_*iz*_ = 0.5 is equivalent to Subject *i* flipping a fair coin to get the *PRO*_*z*_ by pure chance.

As discussed above, based on Expressions (5) and (6), the unbiased estimator of *Q*_*iz*_ is ${\hat{Q}}_{iz}=\max\left\{R_{ij,}j=1,\ldots ,m\right\}$ if *a*_*iz*_ is in the searching set {*a*_*j*,_ *j* = 1, …, *m*}. In practice, many tied maximums of *R*_*ij*_ may occur especially when *t* is small and *m* is large. In this case, the median of the tied maximums will be taken as the estimate. Because of this, ${\hat{Q}}_{iz}$ becomes a consistent estimator. The variance of ${\hat{Q}}_{iz}$ is nuisance because the validation of PROM is usually drawn from multiple subjects instead of Subject *i*. Nonetheless the variance estimate ($\hat{Var}({\hat{Q}}_{iz})$) of ${\hat{Q}}_{iz}$ for Subject *i* can be obtained by $\frac{{\hat{Q}}_{iz}(1-{\hat{Q}}_{iz})}{t}$, because *TP+TN* follows a binomial distribution with parameter *t* and *Q*_*iz*_ when *a*_*j*_ = *a*_*iz*_. Further, because *t* is usually small, the exact binomial confidence interval for *Q*_*iz*_ is used for ${\hat{Q}}_{iz}$ in the simulation study.

It is necessary to point out that if the two probabilities (say *Q*_*_iz*_ for negative truth and *Q*_*+iz*_ for positive truth) are not equal, many more assumptions are needed to estimate *Q*_*_iz*_ and *Q*_*+iz*_. Using our method, when both *Q*_*_iz*_ and *Q*_*+iz*_ are both greater than 0.5 we can have $t[rF_{ik}(a_{iz})+{\bar{F}}_{ik}(a_{iz})]Q_{+iz}=\underset{a_{j}}{Max}(TP+TN)$, where *r* = *Q*_*+iz*_ */Q*_*_iz*_. We can estimate *a*_*iz*_using *a*_*j*_ at which the maximum of (*TP* + *TN*) is reached, but we have unknown *r* and many unknown *F*_*ik*_ (*k* = 1, …, *t*). If we further assume *r* is known, we still could not find the estimate for *Q*_*+iz*_ because we don’t know these *F*_*ik*_. Unless we further assume the distribution function of *X*_*ik*_ at each clinical visit *k*, we can have a consistent estimate of *Q*_*_iz*_ and *Q*_*+iz*_. But we feel that these further assumptions on knowing *r* and *F*_*ik*_ (*k* = 1, …, *t*) are not practical, especially in medical device clinical trials. Therefore, we only search the threshold such that the two probabilities are equal in this paper.

### 2.6 Inference of *Q*_*iz*_ in multiple subjects

So far, *Q*_*iz*_ is estimated based on *t* repeated pairs of measurements from Subject *i* for the PROM_z_. If one wants to know the population *Q*_*z*_ for the *PROM* and the objective measurement *X* at PROM_z_ in a target patient population, the *Q*_*z*_ can be confirmed by the mean ($\sum_{i=1}^{n}{\hat{Q}}_{iz}/n$) of ${\hat{Q}}_{iz}$ with its 95% CI. For example, the lower bound of the 95% confidence interval of *Q*_*z*_ must be greater than a desired probability of revealing truth in order for one to believe that the PROM_z_ is able to reveal disease status for majority of subjects in the patient population.

The ability of the *PROM*_*i*_ to detect a change in the objective endpoint *X*_*i*_ could be confirmed by the statistically significant change of *a*_*iz*_ to *a*_*iz’*_ obtained by different dichotomizations of the *PRO*_*i*_. Note, the magnitude of *a*_*iz*_ will be changed when the *PRO*_*i*_ is dichotomized differently. For example, the *PRO*_*i*_ can be dichotomized at scale 7 by “at least very much improvement or otherwise" or at scale 6 by “at least much improvement or otherwise". This change of dichotomization represents one unit change of the *PRO*_*i*_ from scale 6 to 7, and thus the change of *a*_*i6*_ to *a*_*i7*_ measures the ability of the *PROM*_*i*_ to detect the change in the objective endpoint *X*_*i*_. The *a*_*iz*_ is expected to be larger when the *PRO*_*i*_ is dichotomized by “at least very much improvement or otherwise" compared to that by “at least much improvement or otherwise". This is because “at least very much improvement” is more difficult to be reached and thus its minimum threshold is expected to be higher than that for “at least much improvement”. One can obtain the estimate of the change of *a*_*iz*_ to *a*_*iz’*_ from each of *n* different subjects, and perform the test of the mean change > 0.

## *3* Simulation and illustration

### 3.1 Simulation

Simulation data from Subject *i* is used to illustrate the characteristics of ${\hat{Q}}_{iz}$, especially to show ${\hat{Q}}_{iz}$ is a consistent estimator of *Q*_*iz*_. The simulation is not meant to align with a real clinical trial, however the use of ${\hat{Q}}_{iz}$ in a clinical trial is presented after the simulation using hypothetical clinical data. Because *Q*_*iz*_ is defined at subject level, the simulation uses one treatment for a disease in one subject only. The primary endpoint is an objective endpoint measuring the change of the disease status from baseline to 3 months. The PROM is the 7-scaled disease-related satisfaction PROM such as illustrated in Fig 1. In order to include different means and standard deviations, the simulation uses 5 different means [***μ*** = (0, 0.5, 1, 1.5, 2)] and 5 associated different standard deviations [**σ**^**2**^ = (1, 1.3, 1.6, 1.9, 2.2)] as two building blocks to construct various multivariate normal distribution for the objective endpoint. For example, if *t* = 10 then *X*_*ik*_ (*k* = 1, … 10) will follow the multivariate normal distribution with stacked mean vector (***μ***, ***μ***) and the variance-covariance matrix with diagonal elements of ***σ***^***2***^ repeated similarly on diagonal and the off-diagonal element of *ρσ*_*l*_*σ*_*s*_. Other setups are described as follows:

- The correlation coefficient (*ρ*) between *X*_*ik*_ and *X*_*ik’*_ ranges from 0.3, 0.5, and 0.8.
- ‘*a*_*i7*_’ (the minimum objective threshold for “at least very much improved”) is equal to 1.2, ‘*a*_*i3*_’ is equal to -0.3 and ‘*a*_*i5*_’ is equal to 0.4.
- The underlying probability of revealing the truth, *Q*_*iz*_ (*z* = 3, 5, or 7) has values of 0.5, 0.6, 0.7, 0.8, and 0.9.
- Number of repeated measurements for the subject is *t* = 5, 10, 20, 40.
- Pre-selected *a*_*j*_ ranges from -2 to 5.0 with increasing step of 0.1, therefore *m* = 71. Because the minimum two standard deviations below the five means is -2 and the maximum two standard deviations above the five means is 5, this range is wide enough to include all underlying true values of *a*_*i3*_, *a*_*i5*_, and *a*_*i7*_.
- Number of simulation is 10,000.

For each combination of *ρ* (0.3, 0.5, 0.8), *a*_*iz*_ (-0.3, 0.4, 1.2), and *Q*_*iz*_ (0.5, 0.6, 0.7, 0.8, 0.9)_,_, the *t* (5, 10, 20, 40) pairs of outcomes (*x*_*ik*_, *g*_*ik*_) (*k* = 1, …, *t*) are sampled as follow. First *x*_*ik*_ (*k* = 1, …, *t*) is drawn from the corresponding multivariate normal distribution. If *x*_*ik*_ ≥ *a*_*iz*_, *g*_*ik*_ is drawn from *Bernoulli* (*Q*_*iz*_); otherwise *g*_*ik*_ is drawn from *Bernoulli* (1-*Q*_*iz*_). Then an estimate of *Q*_*iz*_ is calculated using the method described above based on the *t* pairs of outcomes, and its 95% CI is calculated using the exact binomial confidence interval due to small samples. These steps are repeated 10,000 times for each underling value of *Q*_*iz*_ and *t*; and then the mean of these 10,000 ${\hat{Q}}_{iz}$ and the coverage probability of the 95% CIs for the *Q*_*iz*_ are obtained.

**Fig 3:**
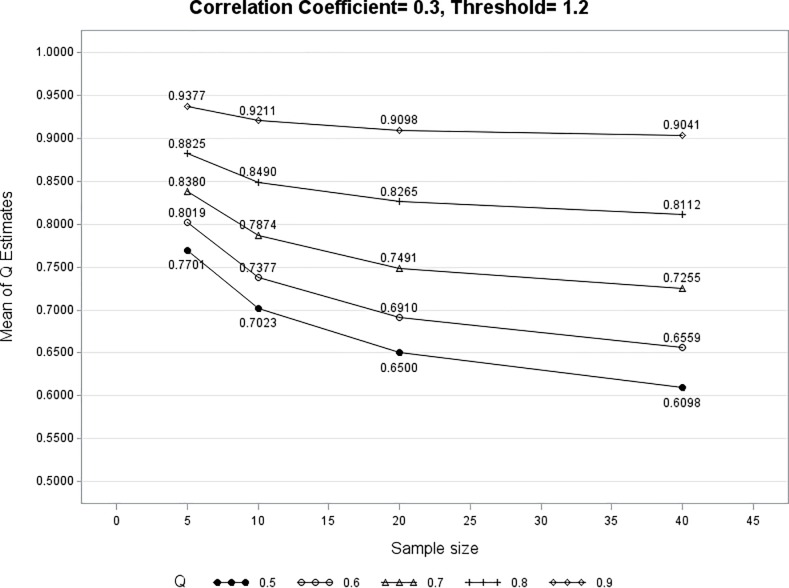
The mean ${\hat{Q}}_{iz}$ converges to its underlying value of *Q*_*iz*_ as sample size increases (*ρ* = 0.3, *a*_*iz*_ = 1.2).

**Fig 4:**
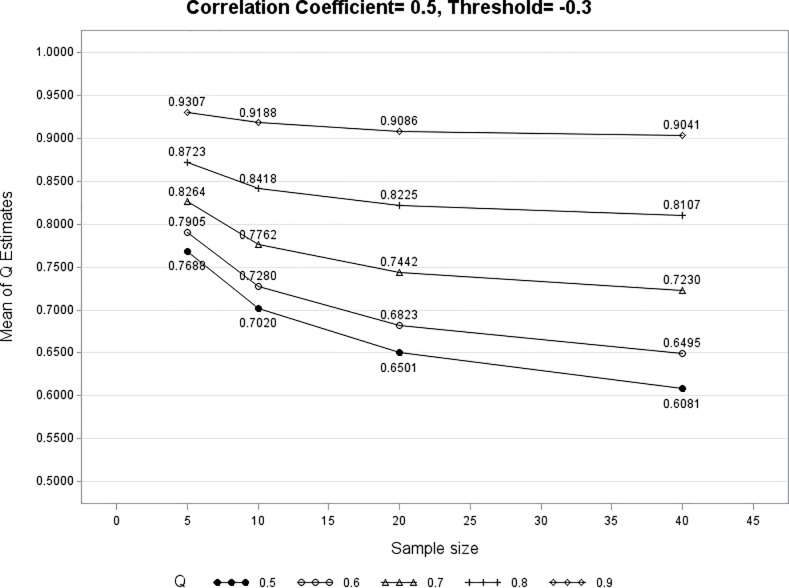
The mean ${\hat{Q}}_{iz}$ converges to its underlying value of *Q*_*iz*_ as sample size increases (*ρ* = 0.5, *a*_*iz*_ = -0.3).

**Fig 5:**
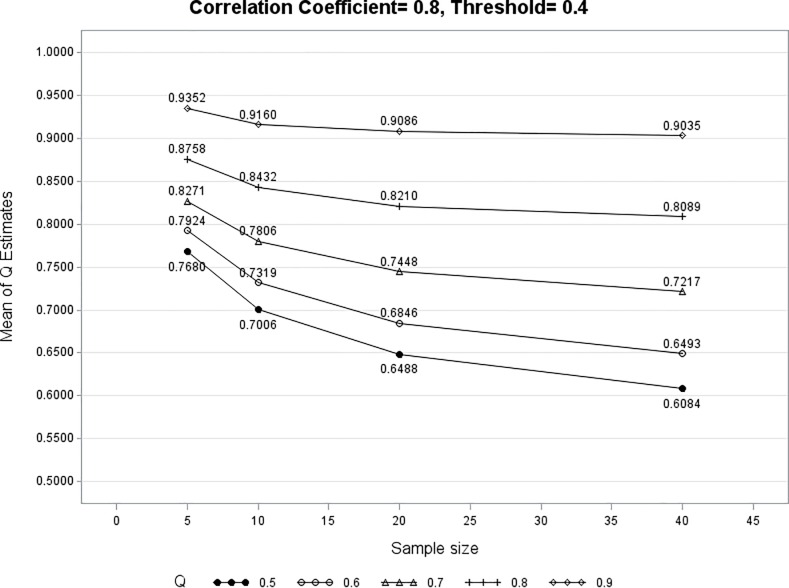
The mean ${\hat{Q}}_{iz}$ converges to its underlying value of Qiz as sample size increases (*ρ* = 0.8, aiz = 0.4).

Figs 3–5 show three examples that the mean of these 10,000 ${\hat{Q}}_{iz}$ converges to *Q*_*iz*_ regardless of the values of *ρ* and *a*_*iz*_. As the number of clinical visits increases for Subject *i*, the mean of ${\hat{Q}}_{iz}$ approaches its underlying true value of *Q*_*iz*_. The converging pattern exists for every value of *Q*_*iz*_ (0.6, 0.7, 0.8, 0.9) except for *Q*_*iz*_ = 0.5. This is not a surprise because when *Q*_*iz*_ = 0.5 there is no association between *PROM*_*i*_ and *X*_*i*_ at PROM_z_. As shown in expressions (5) and (6), when *Q*_*iz*_ = 0.5 every *R*_*ij*_ (*j* = 1 … *m*) is an unbiased estimator of *Q*_*iz*_. A separate simulation using the median of *R*_*ij*_ as ${\hat{Q}}_{iz}$ is performed when *Q*_*iz*_ = 0.5. The mean ${\hat{Q}}_{iz}$ ranges from 0.50 to 0.52 (converging to 0.5) for different combinations of *ρ*, *a*_*iz*_, *Q*_*iz*_, and *t*. In practice, the simulation results for *Q*_*iz*_ = 0.5 in Figs 3–5 can be used as a reference to set a minimum acceptable *Q*_*iz*_ value. Table 3 shows that mean ${\hat{Q}}_{i7}$ is a fairly close estimate of *Q*_*i7*_ under different values of *t* (5, 10, 20, 40). It is found that the probability of the 95% CI including the true value of *Q*_*i7*_ (coverage probability) is at least 95% due to the use of exact binomial confidence interval.

**Table 3:** Mean estimate and coverage probability of *Q*_*i*7_ *ρ* = 0.8, *a*_*i*7_ = 1.2.

		True value of *Q*				
		0.5	0.6	0.7	0.8	0.9
*t* = 40	$\hat{Q}$	0.61	0.65	0.72	0.81	0.90
	Coverage Probability of the 95% CI	0.851	0.958	0.961	0.981	0.971
*t* = 20	$\hat{Q}$	0.65	0.69	0.75	0.82	0.91
	Coverage Probability of the 95% CI	0.841	0.949	0.986	0.982	0.994
*t* = 10	$\hat{Q}$	0.70	0.73	0.78	0.84	0.92
	Coverage Probability of the 95% CI	0.915	0.980	1.000	1.000	0.996
*t* = 5	$\hat{Q}$	0.77	0.79	0.83	0.88	0.94
	Coverage Probability of the 95% CI	1.000	1.000	1.000	1.000	1.000

### 3.2 Case study: Hypothetical clinical trial data

The probability *Q*_*iz*_ of revealing truth for Subject *i* at PROM_z_, has been applied to hypothetical clinical trial data in order to assess the conditional association parameter in multiple subjects. The purpose of the trial is to improve near vision by a medical device. Each subject had a test device implanted and was followed up at Months 3, 6, 12, 18, 24, 30 post procedure. At each follow-up visit, a subject had his/her uncorrected near visual acuity (UCNVA) measured using ETDRS Chart at 40 cm/16 in, and answered a unidimensional PROM question with 7 possible outcomes as shown in Fig 1. The question in the PROM was “*How satisfied are you with your near vision without reading glasses after the treatment*?” The change from baseline in UCNVA is considered as the continuous objective clinical endpoint with a larger change indicating better near vision. The outcome of the satisfaction question is the *PRO* which can be dichotomized in 3 ways for every subject: ≥5 or otherwise, ≥6 or otherwise, ≥7 or otherwise. The mean ${\hat{Q}}_{iz}$ (*z* = 5, 6, or 7) is used to assess the probability of the PROM_z_ to reveal the status of the visual acuity in the targeted population.

The pre-determined threshold searching set {*a*_*j*,_ *j* = 1, …, *m*} ranges from -20 to 60 letters with an increasing step of 1. This set contains *m* = 81 searching points for the minimum threshold *a*_*iz*_ (*z* = 5, 6, or 7). It is believed that the threshold-searching set is large enough to contain the true value of *a*_*iz*_ for PROM_z_ for every subject in the target population.

**Table 4** below shows that the mean of the ${\hat{Q}}_{iz}$ (probability of revealing truth) and the mean ${\hat{a}}_{iz}$ in the change of UCNVA. As expected, one can see that the highest satisfaction has the lowest mean probability of revealing truth uncorrected visual acuity and the largest threshold in the change of UCNVA: 21 more letters correctly identified from baseline. The associated 95% CIs for *Q*_*iz*_ well exclude 0.5 indicating *Q*_*iz*_ from the majority of subjects are greater than 0.5 and consequently the probability of the PROM_z_ revealing subjects’ uncorrected visual acuity is established. Since the PROM_z_ has > 83% probability (based on the lower limits) of revealing the status of UCNVA, it may be used as a binary endpoint for the primary inference for uncorrected near visual acuity.

**Table 4:** Mean ${\hat{Q}}_{iz}$ and mean ${\hat{a}}_{iz}$ in the change of UCNVA.

Satisfaction dichotomized value	# of subjects *	Mean ${\hat{Q}}_{iz}$ (97.5% CI)	Mean ${\hat{a}}_{iz}$ (# letters correctly identified)
≥5	414	0.91 (0.893, 0.917)	8
≥6	324	0.88 (0.864, 0.893)	14
≥7	190	0.85 (0.831, 0.868)	21

* includes subjects whose PROs contain the dichotomized value and have at least two different objective outcomes

**Table 5** shows the median of ${\hat{a}}_{iz}‑{\hat{a}}_{iz\prime }$ when the satisfaction level changes. The ${\hat{a}}_{iz}‑{\hat{a}}_{iz\prime }$ is found to have a highly skewed distribution; therefore p-values are reported here from a non-parametric signed rank test, and the reference statistic is referred to median instead of mean. One can observe that

1. When the *PRO* increases from ≥5 to ≥6, the majority of subjects have no change (median = 0) in their uncorrected near vision acuity; this means that the *PRO* change from scale 5 to scale 6 may not represent a change in majority subjects’ uncorrected near vision acuity.
2. When the *PRO* increases from ≥6 to ≥7 or ≥5 to ≥7, the majority of subjects have a positive change (median = 9 or 21, respectively) in their uncorrected near vision acuity; this means that the *PRO* changes from a lower score to 7 represent a change in majority subjects’ uncorrected near vision acuity.

**Table 5:** Ability of detecting a change: Median of ${\hat{a}}_{iz}‑{\hat{a}}_{iz\prime }$.

Satisfaction Change	# of subjects *	change of ${\hat{a}}_{iz}‑{\hat{a}}_{iz\prime }$		p-value by Signed Rank Test
		Mean	Median	
From ≥5 to ≥6	324	11	0	<0.001
From ≥6 to = 7	190	15	9	<0.001
From ≥5 to = 7	190	20	21	<0.001

*includes all subjects who are in Table 3 and have a change objective value when the associated PRO changes

These indicate that a change of one PROM unit in this case might not be adequate for a translation to a change in uncorrected near visual acuity. An increase of at least two (2) PROM units represents that the majority subjects have a positive increase in their uncorrected near visual acuity. Consequently, the ability of detecting the change of uncorrected near vision function by this PROM is suggested by two (2) PROM units in this clinical trial instead of one (1) PROM unit; or the majority of subjects have their PRO scores changed to 7. It is noted that the number of samples from each subject is ≤ 6 in this trial, which limits the capability of this method to search for *a*_*iz*_.

## *4* Concluding remarks

The conditional probability *Q*_*iz*_ revealing the true status of Subject *i*’s disease at PROM_z_ is a new quantitative statistic assessing the conditional association between a unidimensional *PROM*_*i*_ and a continuous objective endpoint *X*_*i*_ measuring the disease status. The probability *Q*_*iz*_ of revealing truth is estimated for each subject using paired observations (*x*_*ik*_, *g*_*ik*_) measured repeatedly at different clinical visits (such as Months 3, 6, 12 etc.). The *Q*_*iz*_ reveals truth with respect to the latent minimum objective threshold *a*_*iz*_ (i.e. *x*_*ik*_ ≥ *a*_*iz*_, or *x*_*ik*_ < *a*_*iz*_). When the *PROM*_*i*_ doesn’t associate with the objective endpoint *X*_*i*_, the *Q*_*iz*_ is equal to the pure chance of 0.5. Because *Q*_*iz*_ is a probability measure, this situation looks like one has flipped a fair coin to get his/her *PRO* regardless the status of his/her disease. When a PROM is used as a measure for a disease/condition in a clinical trial setup, the probability of revealing truth must be at least statistically greater than the pure chance of 0.5.

The threshold searching set {*a*_*j*_: *j* = 1, …, *m*} can be pre-determined using the current clinical standard of the possible minimum and maximum objective measurements in the target population. For example, the human hemoglobin concentration ranges from 5 g/dL to 20 g/dL. The number *m* can be determined based on how precise *a*_*iz*_ is expected to be.

In practice, a clinical trial has *n* subjects and thus has *n* estimates of *Q*_*iz*_ (*i* = 1, …, *n*). In order to have the PROM_z_ used for a target population, the majority of *Q*_*iz*_ (*i* = 1, …, *n*) have to be greater than the pure chance of 0.5; or it is equivalent to say that the mean/median of the *Q*_*iz*_ (*i* = 1, …, *n*) should be greater than 0.5. Although the mean/median of the *Q*_*iz*_ > 0.5 would indicate some association between the *PROM* and the objective endpoint *X* greater than chance in the target population, a higher quality *PROM* should have a larger value of the mean/median of the *Q*_*iz*_ (*i* = 1, …, *n*). Let’s denote *δ* as the minimum value of the mean/median of the *Q*_*iz*_ (*i* = 1, …, *n*) which is an acceptable probability for PROM_z_ to reveal the status of the majority of subjects’ disease. To confirm that the majority of subjects have their *Q*_*iz*_ (*i* = 1, …, *n*) greater than *δ*, one can simply test that the mean/median of the *Q*_*iz*_ (*i* = 1, …, *n*) among *n* different subjects is >*δ*.

When the *PRO* is dichotomized differently by one PROM unit increased at a time, one can get the associated estimate of the change of the minimum threshold in the objective measurement for each subject, such as ${\hat{a}}_{iz}-{\hat{a}}_{iz\prime }$ (*i* = 1, …, *n*). If the mean of these estimates from different subjects is statistically significantly greater than 0, then the PROM has the ability to detect a change in the objective endpoint. In case that ${\hat{a}}_{iz}-{\hat{a}}_{iz\prime }$ (*i* = 1, …, *n*) has a skewed distribution, one should use the median of the estimates of ${\hat{a}}_{iz}-{\hat{a}}_{iz\prime }$ (*i* = 1, …, *n*) so that the test implies that the majority of *a*_*iz*_ − *a*_*iz*′_ (*i* = 1, …, *n*) are greater than 0.

The limitations of using *Q*_*iz*_ include (1) it is applicable to a unidimensional PROM or a PROM item of interest in a multi-dimensional PROM instrument when a valid continuous objective measure of the disease status exists, and (2) if the number of repeated measurements is small, the estimator of *Q*_*iz*_ is more biased. In this case, one can adjust the minimum acceptable probability of revealing truth in order to have confidence for the PROM_z_ to reveal truth. Further research may focus on a quantitative method for measuring the conditional association between a multi-dimensional PROM and a pertinent objective measurement.

## Appendix A: Notations

- Sub-indexes *i* and *j* represent Subject *i* and threshold searching point *j* within a clinical visit *k* (*i* = 1, …, n, *j* = 1, …, m, and *k* = 1, …, t). The letter *z* denotes the z^th^ scale of the PROM (PROM_z_).
- The *a*_*iz*_ is a fixed parameter which is defined as the minimum latent threshold in terms of the objective measurement for Subject *i* at PROM_z_. The *a*_*iz*_ is defined for the z^th^ scale and Subject *i*. For example, if the PROM has 5 different scales, then we will have five different values of *a*_*iz*_ for the subject.
- The *a*_*j*_ is the *j*^th^ searching point for *a*_*iz*_, and the *a*_*j*_ belongs to a fixed pre-selected threshold searching set {*a*_*j*_: *j* = 1, …, m} (such as the normal range of hemoglobin count with an increasing step of 0.5). The *a*_*j*_ is a nonrandom variable and does not change with subject. The set is selected based on the current clinical standard of normal range.
- The *X* is the random variable for the continuous objective measurement of the status of a subject’s disease/condition, and lower case *x* is an outcome/realization of *X*.
- $G_{ik}^{1}$ is the Bernoulli random variable with probability *Q*_*iz*_ to be 1 when *x*_*ik*_ ≥ *a*_*iz*_.
- $G_{ik}^{0}$ is the Bernoulli random variable also with probability *Q*_*iz*_ to be 0 when *x*_*ik*_ < *a*_*iz*_.
- *G*_*ik*_ represents two mixed Bernoulli random variables with the same parameter *Q*_*iz*_ (but opposite meaning) $G_{ik}^{1}$ (if *x*_*ik*_ ≥ *a*_*iz*_) or $G_{ik}^{0}$ (if *x*_*ik*_ < *a*_*iz*_).
